# SDA-YOLO: An Object Detection Method for Peach Fruits in Complex Orchard Environments

**DOI:** 10.3390/s25144457

**Published:** 2025-07-17

**Authors:** Xudong Lin, Dehao Liao, Zhiguo Du, Bin Wen, Zhihui Wu, Xianzhi Tu

**Affiliations:** 1 College of Mathematics and Informatics, South China Agricultural University, Guangzhou 510640, China; hunanlxd@163.com (X.L.); 15970544033@163.com (D.L.); wenzip@scau.edu.cn (B.W.); wuzhihui@scau.edu.cn (Z.W.); 2 College of Arts, South China Agricultural University, Guangzhou 510640, China; xianzhitu@scau.edu.cn

**Keywords:** YOLO, peach, multi-scale feature fusion, object detection

## Abstract

**Highlights:**

**What are the main findings?**

- This study proposes SDA-YOLO, an enhanced peach detection framework based on YOLOv11n, which introduces an innovative Adaptive Multi-Scale Fusion Pyramid (AMFP) module. The AMFP module combines adaptive mechanisms with multi-scale feature fusion and pyramid architecture, dynamically optimizing fusion strategies across hierarchical features (high-, medium-, and low-resolution). This effectively mitigates the inflexibility of conventional feature fusion methods when handling scale variations induced by peach occlusion and illumination heterogeneity, thereby substantially improving the model’s capacity to process complex feature representations.
- The following three key enhancements are implemented in the network architecture: (1) integration of Large Kernel Attention (LSKA) mechanisms into the backbone network to construct SPPF-LSKA fusion modules; (2) replacement of CIoU with MPDIoU-based bounding box regression loss functions; and (3) incorporation of Dynamic Head (DyHead) blocks to form DMDetect modules. These synergistic improvements collectively enhance detection robustness in challenging orchard environments.

**What is the implication of the main finding?**

- The AMFP module presents a novel solution to multi-scale feature fusion challenges in object detection, particularly addressing issues arising from occlusion and illumination variance. Its adaptive fusion strategy adjustment capability demonstrates superior performance, not only in peach detection tasks, but it also provides methodological references for object detection in analogous complex scenarios. This advancement holds the potential to accelerate the development of detection technologies in agricultural applications and related domains.
- The comprehensive architectural improvements in SDA-YOLO achieve significant performance gains in peach detection accuracy and reliability under complex orchard conditions. For agricultural vision tasks characterized by dense occlusions and cluttered backgrounds, this framework establishes a robust technical foundation for intelligent fruit recognition, automated harvesting systems, and yield estimation. These advancements contribute to enhanced automation in orchard management, reduced labor costs, and improved economic efficiency in agricultural production systems.

**Abstract:**

To address the challenges of leaf–branch occlusion, fruit mutual occlusion, complex background interference, and scale variations in peach detection within complex orchard environments, this study proposes an improved YOLOv11n-based peach detection method named SDA-YOLO. First, in the backbone network, the LSKA module is embedded into the SPPF module to construct an SPPF-LSKA fusion module, enhancing multi-scale feature representation for peach targets. Second, an MPDIoU-based bounding box regression loss function replaces CIoU to improve localization accuracy for overlapping and occluded peaches. The DyHead Block is integrated into the detection head to form a DMDetect module, strengthening feature discrimination for small and occluded targets in complex backgrounds. To address insufficient feature fusion flexibility caused by scale variations from occlusion and illumination differences in multi-scale peach detection, a novel Adaptive Multi-Scale Fusion Pyramid (AMFP) module is proposed to enhance the neck network, improving flexibility in processing complex features. Experimental results demonstrate that SDA-YOLO achieves precision (P), recall (R), mAP@0.95, and mAP@0.5:0.95 of 90.8%, 85.4%, 90%, and 62.7%, respectively, surpassing YOLOv11n by 2.7%, 4.8%, 2.7%, and 7.2%. This verifies the method’s robustness in complex orchard environments and provides effective technical support for intelligent fruit harvesting and yield estimation.

## 1. Introduction

Peaches are favored by consumers for their nutritional components and delicious taste [1]. In recent years, the rapid growth of the global population has led to a surging demand for fruits [2]. The fruit industry has been growing rapidly, with peach cultivation significantly expanding, making peaches the third most popular temperate fruit after apples and pears [3,4]. In practical scenarios, fruit counting enables growers to optimize decisions regarding purchasing bagging materials and hiring workers [5]. Furthermore, these data can significantly improve peach yield and quality while optimizing orchard management. However, due to the complexity of orchard environments, fruit counting and harvesting mostly remain at manual counting stages [6], which is time-consuming, labor-intensive, and inefficient. Particularly during harvest seasons, the need to pick large quantities of peaches within short periods creates substantial labor demands [7]. Therefore, traditional manual picking methods struggle to meet efficient and precise peach harvesting requirements. There is an urgent need for detection algorithms that can effectively identify peach fruits to provide technical support for intelligent picking.

However, accurate fruit recognition in complex environments remains a core technical bottleneck restricting agricultural intelligence development. Specific challenges include the following: complex canopy structures and uneven fruit distribution in peach trees, compounded by factors like uneven illumination and occlusion; significant image brightness and color distortion caused by natural light variations; loss of key feature information due to mutual occlusion between fruits or coverage by leaves and branches; notable color and shape differences among fruits of different varieties and maturity levels; and interference from complex backgrounds, including soil, weeds, and branches that further reduce recognition accuracy.

With iterative upgrades in deep learning technology, fruit detection based on deep learning is undergoing technological innovation. Deep neural networks leverage their powerful feature learning capabilities to automatically extract robust visual features under varying illumination, significantly reducing dependence on image preprocessing. Supported by large-scale cross-scenario dataset construction, models demonstrate exceptional generalization performance in multi-variety fruit recognition tasks. The introduction of dynamic instance segmentation technology effectively addresses mutual occlusion between fruits while enhancing key region perception through attention mechanisms. Optimized lightweight network architectures achieve real-time detection capabilities in complex field backgrounds, meeting diverse scenario and task requirements.

Convolutional neural network-based fruit detection methods have achieved significant breakthroughs in accuracy and efficiency, effectively extracting fruit visual features in orchard environments. However, their detection performance still shows obvious limitations when facing complex agricultural scenarios involving leaf occlusion, fruit overlap, and multi-scale variations. Particularly, in peach detection tasks, traditional methods often suffer from feature confusion due to high textural similarity and dense distribution characteristics of targets. Existing models also exhibit insufficient feature representation capabilities for small-scale targets and partially occluded fruits, severely constraining the accuracy of practical applications like picking robots. To address these challenges, this study proposes an improved SDA-YOLO detection model, with core innovative contributions as follows:

1. Multi-scale Feature Enhancement Module: Integrates Large Kernel Attention (LSKA) into the standard SPPF module to construct a SPPF-LSKA fusion structure. By leveraging adaptive receptive field adjustment strategies and the spatial information capture capability of large kernel convolution, this module enhances the model’s feature representation ability for multi-scale peach targets and improves feature extraction effectiveness across different scales.

2. Bounding Box Regression Loss Function Optimization: Replaces CIoU with MPDIoU-based regression loss function. Through joint optimization of center point distance and aspect ratio, this approach effectively mitigates localization deviations caused by leaf occlusion, significantly improves spatial distinction between overlapping fruits, and enhances positioning accuracy for occluded peaches.

3. Detection Head Improvement: Integrates DyHead Block into the detection head to construct a DMDetect module. By leveraging the synergistic effects of scale perception and spatial attention mechanisms, this module strengthens feature responses for small targets and occluded regions in complex backgrounds, reduces false detection/missing detection caused by branch interference, and enhances feature discrimination capability for small and occluded targets in complex environments.

4. AMFP (Adaptive Multi-scale Fusion Pyramid) Module: Combines adaptive mechanisms with multi-scale feature fusion and pyramid structures. Through dynamic adjustment of fusion strategies for different hierarchical features (high-, medium-, and low-resolution), this module enhances model flexibility in processing complex features, improves adaptability to complex scenes, and addresses insufficient feature fusion flexibility caused by scale variations from peach occlusion and illumination differences.

## 2. Related Works

In fruit target detection, early research primarily used traditional image processing and machine learning with manually designed features like color, shape, and texture. In 2010, Li et al. [8] developed a real-time recognition system for pineapple harvesting, detecting fruits like tomatoes and apples using color segmentation with 88.0% accuracy. Wei et al. [9] achieved 95% extraction accuracy via threshold segmentation in complex backgrounds (2014). Zhuang et al. [10] combined adaptive red–green maps, Otsu thresholding, and SVM for citrus detection, achieving >0.86 recall (2018). Syazwani et al. [11] used ANN, SVM, and RF with shape/color/texture features for pineapple crown classification, attaining 94% accuracy (2022). While partially effective, these methods had inflexible feature selection and heavy reliance on manual feature engineering, leading to an increased focus on deep learning.

With the advancement of deep learning, object detection algorithms have evolved into one-stage and two-stage frameworks. Two-stage detectors, represented by Faster R-CNN, improve accuracy via region proposals. Gao et al. [12] used Faster R-CNN in 2020 for multi-category apple detection, achieving mAPs of 0.909 (unobstructed), 0.899 (leaf-occluded), 0.858 (branch-occluded), and 0.848 (fruit-occluded). Basli et al. [13] employed Faster R-CNN with MobileNet for mango and dragon fruit detection/classification, attaining near 99% accuracy. Jia et al. [14] proposed an optimized Mask R-CNN in 2022, incorporating MobileNetv3 and a boundary patch module, achieving 76.3% mAP and 81.1% mAR on persimmon dataset improvements of 3.1% and 3.7% over baseline.

Among one-stage object detection algorithms, the YOLO series has gained extensive application in orchard fruit detection due to its efficient detection pipeline. Song et al. [15] improved YOLOv3 in 2021 by replacing the last three downsampling layers of Darknet53 with DenseNet’s dense connection mechanism, achieving 80.98% mAP for immature citrus recognition. Hao et al. [16] proposed a hybrid data augmentation method in 2022, substituting YOLOv3’s backbone with MobileNet-v3 to elevate the green walnut detection mAP to 94.52%. In the same year, Song et al. [17] enhanced YOLOv5 for oil fruit detection in natural environments, attaining 98.71% mAP; Zhang et al. [18] incorporated a transformer module with attention mechanisms into YOLOv5, improving cherry fruit detection mAP by 3.77%; Lv et al. [19] achieved mAP values of 77.4% and 53.5% for citrus and branch detection, respectively, through stripe attention modules in YOLOv5’s backbone and using semi-supervised methods; Xie et al. [20] boosted the litchi dataset mAP by 12.9% via attention modules and improved loss functions in YOLOv5.

Researchers continue to optimize YOLO models for diverse fruit species and detection scenarios. Tang [21] developed YOLOv7-Plum in 2023 for natural environment plum detection; another team proposed a YOLOv7-based yellow peach detection solution the same year, achieving an 80.4% detection mAP through CA attention mechanisms and modified loss functions [22]. Li et al. [23] proposed PeachYOLO in 2024 for peach detection in complex orchard environments, employing partial convolutions and Channel Attention (CA) mechanisms to enhance detection heads, while incorporating Deformable Convolutions (DCNv2) to improve model robustness. Additionally, Zhang et al. [24] developed a YOLOX-based fruit counting algorithm, achieving accurate Holly fruit counts through scenario-specific sample augmentation; Fu et al. [25] proposed YOLOv5-AT with data augmentation techniques, attaining mAP values of 84.6%, 98.0%, and 85.1% for green oranges, green tomatoes, and green persimmons, respectively, in color-similar backgrounds.

Regarding multi-scale feature fusion and complex environment adaptability, Nan et al. [26] designed the WGB-YOLO network, improving dragon fruit detection accuracy in densely planted orchards through WFE-C4 and GF-SPP modules for enhanced cross-channel and spatial feature extraction; Zhu et al. [27] proposed the lightweight YOLO-LM model, addressing occlusion and scale inconsistency in oil tea fruit detection via cross-attention and Adaptive Spatial Feature Fusion (ASFF) modules, achieving 93.96% precision, 93.32% recall, and 93.18% mAP@0.5. Xu et al. [28] developed the EMA-YOLO model for immature yellow peach detection, achieving a 4.2% mAP improvement over the YOLOv8n baseline through integrated attention mechanisms and EIoU loss functions, providing an effective solution for small target detection.

These studies demonstrate the effectiveness of YOLO algorithms in orchard fruit detection. However, in peach fruit detection tasks within complex orchard environments characterized by variable backgrounds, small fruit sizes, and severe foliage occlusion, the original YOLO networks face significant challenges. Therefore, further improvements are necessary to enhance target detection capabilities.

## 3. Materials and Methods

### 3.1. Dataset Acquisition and Data Augmentation

**Figure 1:**
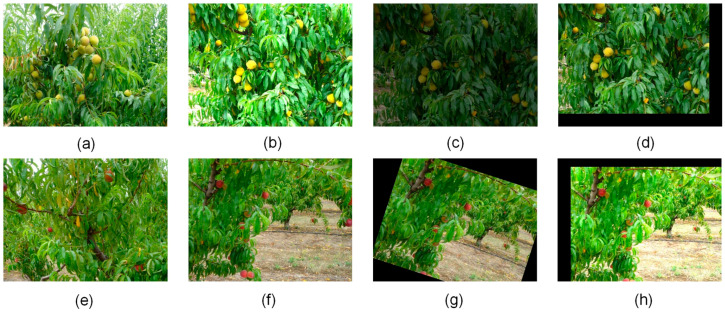
Partial data image examples: (**a**) original image; (**b**) increased brightness; (**c**) reduced brightness; (**d**) translation; (**e**) original image; (**f**) noise addition; (**g**) rotation; (**h**) brightness change and translation.

The dataset originates from publicly available online resources and is jointly developed by the research team of Beiran University in Portugal and the Polytechnic Institute of Castelo Branco, designed for peach detection research. The dataset includes images of the following three peach varieties: Catherine (381 images), Royal Time (311 images), and Sweet Dream (336 images). Each image has a resolution of 640 × 480 pixels, and the dataset comprises a total of 1028 original images. The dataset is divided into training, validation, and test sets in a ratio of 7:2:1, specifically comprising 739 training images, 192 validation images, and 97 test images. To enhance the model’s generalization ability and robustness, data augmentation strategies have been applied to the training set. Through operations including rotation (±15 degrees), shear (horizontal and vertical, ±20 degrees), blurring (maximum kernel size 2.5 pixels), the addition of salt-and-pepper noise (up to 0.1% density), horizontal and vertical flipping, and adjustments to hue (±15), saturation (±25), and brightness (±15), the training set was expanded to 4032 images, resulting in an augmented dataset totaling 4321 images, with a total of 26,089 annotated bounding boxes. These augmentation parameters were selected to simulate realistic variations encountered in orchard environments during automatic harvesting and UAV-based yield estimation under normal weather conditions, such as minor viewpoint changes, illumination differences, subtle occlusions, and sensor noise, while carefully balancing the need for increased robustness against the risk of introducing excessive distortion that could compromise the localization accuracy essential for these applications. Examples of the augmented images after the above-mentioned operations are shown in Figure 1. The number of images and the number of peach fruits in each dataset are shown in Table 1.

### 3.2. Methods

#### 3.2.1. YOLOv11

YOLOv11 is the latest version in the YOLO series of object detection algorithms. Its network architecture inherits the end-to-end, multi-scale detection design philosophy of the YOLO family while introducing optimized upgrades to the backbone network, feature fusion, and detection head modules. The overall framework consists of three core components, which are as follows: backbone, neck, and detection head. The backbone network adopts an improved CSPNeXt architecture, incorporating C3K2 and C2PSA modules. The C3K2 module enhances feature extraction through customized convolutional kernel sizes and channel separation strategies based on CSP blocks. The C2PSA module combines channel attention with multi-head attention mechanisms to strengthen spatial and channel information fusion, while replacing partial traditional convolutions with lightweight modules to reduce parameters and improve complex feature capture capabilities. The neck network builds upon the PAN architecture, integrating SPFF modules and Adaptive Feature Enhancement (AFE) technology. Through parallel processing of spatial context and feature refinement, it reinforces multi-scale feature interaction and semantic consistency. The detection head maintains the efficiency of YOLO series designs while optimizing classification and regression branches. It introduces depth-wise separable convolutions in the classification branch, implements an adaptive anchor box mechanism for automatic anchor configuration optimization, and combines CIoU loss functions to improve localization accuracy. Additionally, it employs a “consistent dual assignment strategy” for NMS-free end-to-end training to reduce redundant detections. Furthermore, YOLOneirv11 enhances training efficiency through mixed-precision training techniques and supports multi-task capabilities with cross-platform deployment, which is compatible with edge devices, cloud systems, and GPU environments. Compared to other detection models, YOLOv11 demonstrates computational efficiency advantages, enabling efficient deployment on resource-constrained devices while meeting real-time detection requirements.

**Figure 2:**
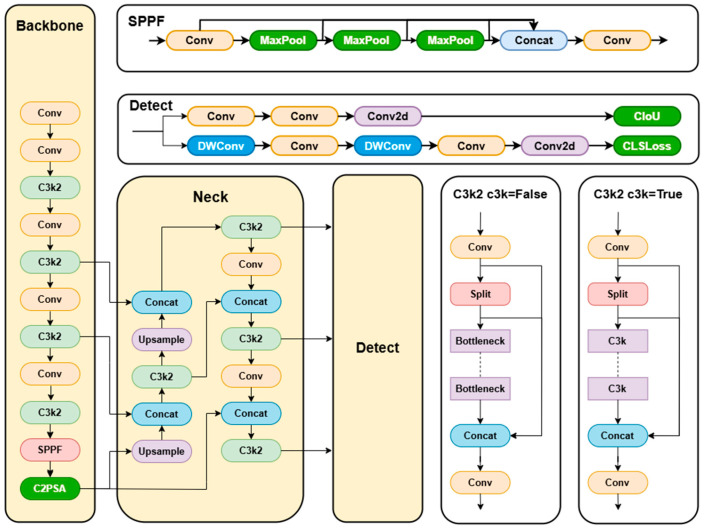
The network architecture of YOLOv11.

**Figure 3:**
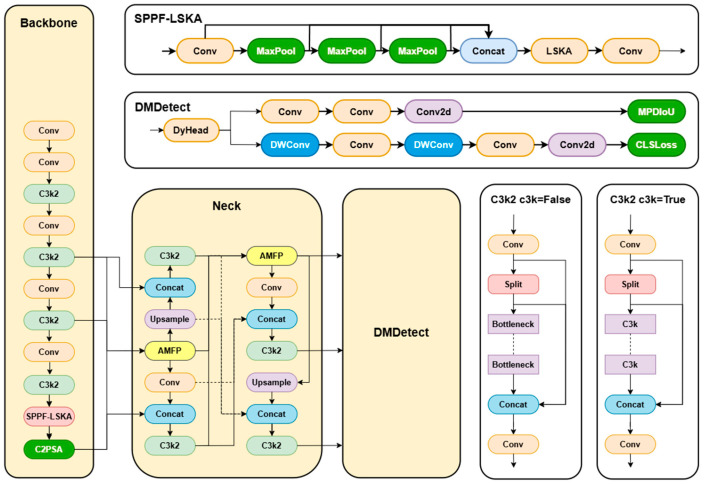
The network architecture of SDA-YOLO.

YOLOv11 comes in the following five sizes: n, s, l, m, and x. The YOLOv11n lightweight model is ideal for edge devices or scenarios demanding high-speed performance. Hence, this study opts for YOLOv11n as the baseline network. This research introduces SDA-YOLO, an improved YOLOv11n-based peach detection method. Firstly, we integrate the Large Kernel Spatial Attention mechanism (LSKA) into the standard SPPF module to form the SPPF-LSKA fusion module. By combining the spatial information capturing ability of large kernel convolution with adaptive receptive field adjustment, this module enhances the feature representation of multi-scale peach targets. Secondly, the MPDIoU-based bounding box regression loss function replaces CIoU. By jointly optimizing the center distance and aspect ratio, it improves the positioning accuracy of overlapping and occluded peaches. In the detection head design, we incorporate the DyHead Block to create the DMDetect module. This module utilizes a multi-branch dynamic detection structure along with scale-awareness and spatial attention mechanisms to strengthen the feature discrimination of small and occluded targets in complex backgrounds. To address the insufficient flexibility of feature fusion caused by scale changes in peaches due to occlusion and lighting differences, we propose the AMFP module. It incorporates an adaptive mechanism into a multi-scale feature fusion pyramid structure, dynamically adjusting the fusion strategy of high-, medium-, and low-resolution features. This enhances the model’s flexibility in handling complex features. The network architecture of YOLOv11 is shown in Figure 2. The network architecture of SDA-YOLO is shown in Figure 3.

#### 3.2.2. SPPF-LSKA

To address the challenges of multi-scale variance in peach fruit detection within complex orchard environments, this study proposes an SPPF-LSKA fusion module integrated into the backbone network of YOLOv11n. Traditional large kernel convolution operations in multi-scale feature extraction face a dilemma between capturing long-range dependencies and computational efficiency, while fixed receptive field mechanisms struggle to adapt to diverse scenarios such as dense foliage occlusion and strong background interference. The LSKA [29] module decouples traditional large kernel convolution operations into cascaded horizontal (1 × 3) and vertical (3 × 1) separable 1D convolutions, effectively preserving the long-range dependency capture capability of large kernel convolution while significantly reducing computational complexity. The module incorporates a dynamically adjustable dilation rate mechanism; for instance, in dense foliage areas, the dilation rate is increased to enable a 3 × 3 convolutional kernel to equivalently cover the receptive field of a 5 × 5 region, thereby capturing edge features of partially occluded fruits. In regions with strong background interference, the dilation rate is appropriately reduced to focus on local details and suppress noise interference from leaf textures.

**Figure 4:**
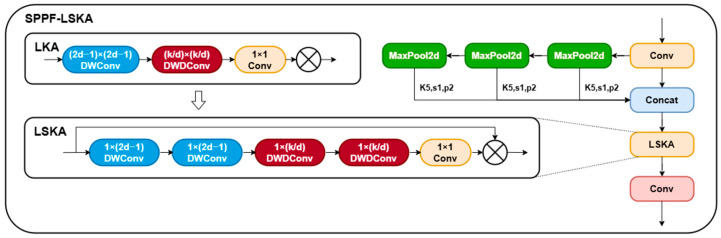
SPPF-LSKA Module Structure Diagram. ⊗: represents Hadamard product. k: represents the maximum receptive field. d: represents the dilation rate.

During the feature fusion stage, SPPF-LSKA generates the original features and three-pooled features at different scales through spatial pyramid pooling, combined with spatial attention maps from LSKA for dynamic weighting of multi-level features. Unlike traditional SPPF that relies on single max-pooling operations, causing detail loss and adopting static fusion strategies insufficient for complex illumination variations, this design achieves refined screening of multi-scale features through lightweight attention mechanisms. Compared to traditional SPPF that relies on single max-pooling operations causing detail loss, this design achieves refined screening of multi-scale features through lightweight attention mechanisms. Under complex orchard illumination conditions (e.g., strong reflections and shadow alternations), it effectively balances local feature extraction in leaf-occluded regions with global contextual associations. Experimental validation demonstrates that this module significantly enhances feature representation capabilities for small-scale fruits in dense foliage occlusion scenarios while maintaining model lightweight characteristics, providing an efficient and robust visual perception foundation for agricultural harvesting robots. The network structure of the improved SPPF-LSKA is shown in Figure 4.

#### 3.2.3. DMDetect

DMDetect is an improved dynamic multi-branch detection module based on the YOLOv11n detection head, which addresses the challenges of peach fruit detection in complex orchard environments—including mutual occlusion between leaves/branches and fruits, fruit stacking, and small-scale partial fruits—by integrating DyHead Block [30] to construct a multi-branch dynamic detection structure. The original YOLO detection head relies on fixed anchor mechanisms and single-scale feature fusion, which are inherently limited in adapting to the variable scales and occluded states of peaches in complex backgrounds. Such static strategies often fail to effectively extract discriminative features for small-scale or occluded fruits, where target boundaries are ambiguous and contextual information is critical for accurate recognition. To resolve this, DMDetect introduces DyHead Block that combines scale-aware and spatial attention mechanisms to enhance the expressive capability of target features in complex backgrounds.

**Figure 5:**
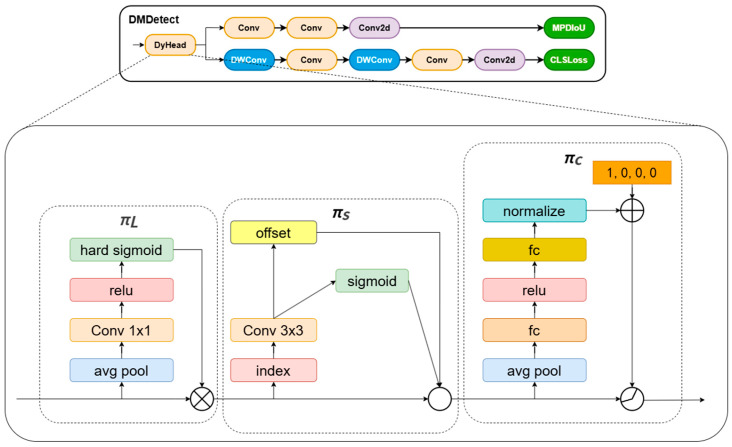
DMDetect Module Structure Diagram. $\pi_{L}$: scale-aware attention. $\pi_{S}$: space-aware attention. $\pi_{C}$: task-aware attention.

The core improvements of DMDetect lie in its dynamic multi-branch structure and attention mechanism design, which directly address the rigidity of traditional detection heads. The DyHead Block adaptively adjusts convolutional kernel receptive fields through dynamic deformable convolution (DyDCNv2), enabling the model to dynamically expand or contract the feature extraction range according to the spatial distribution of occluded or small fruits. In contrast to conventional fixed-kernel convolutions that may either overlook fine-grained details (in large kernels) or miss global context (in small kernels), this adaptive mechanism ensures robust feature capture for both partially visible fruits and distant small targets. Its spatial attention module employs a deformable convolution and mask generation mechanism (mask_dim = 1 × 3 × 3) to strengthen the edge and texture feature extraction of fruits while suppressing leaf and background noise interference, which is particularly effective in scenarios where fruits are partially hidden by overlapping branches. The scale-aware module dynamically fuses features from different levels through adaptive average pooling and global context weighting, improving model sensitivity to small-scale fruits (e.g., distant or occluded peaches) by emphasizing scale-specific semantic information that static fusion strategies might ignore. This dynamic architecture breaks away from the fixed feature processing pipeline of traditional detection heads, allowing end-to-end learning to adaptively adjust contributions from different attention mechanisms based on the complexity of input scenes, thus demonstrating stronger feature discriminability in occluded and multi-scale conditions. The improved DMDetect module structure is illustrated in Figure 5.

#### 3.2.4. MPDIoU

CIoU is the bounding box regression loss function in YOLOv11. It adds center point distance and aspect ratio penalty items to refine the traditional IoU. Its formula is as follows:

(1) $$\mathrm{CIoU}=\mathrm{IoU}-\frac{\rho^{2}}{c^{2}}-\alpha v$$

In the formula, $\rho^{2}={\left(x_{1}-x_{2}\right)}^{2}+{\left(y_{1}-y_{2}\right)}^{2}$ denotes the squared Euclidean distance between the center points of the predicted and ground truth boxes. c represents the diagonal length of the smallest enclosing rectangle that encompasses both boxes. $v=\frac{4}{\pi^{2}}{\left(\arctan\frac{w_{2}}{h_{2}}-\arctan\frac{w_{1}}{h_{1}}\right)}^{2}$ quantifies the consistency of the aspect ratio. $\alpha =\frac{v}{1-IoU+v}$ serves as an adaptive weighting factor. Even though CIoU enhances regression by imposing multi-dimensional constraints, it still has limitations in scenarios involving overlaps or occlusions. To begin with, when the aspect ratios of the predicted and ground truth boxes draw close, the gradient of the aspect ratio penalty term v diminishes, thereby stalling the optimization process. Furthermore, in situations where there is a high degree of overlap, the room for optimizing the center point distance $\rho^{2}$ becomes restricted, making it difficult to distinguish minor differences in the position of bounding boxes.

In order to address these issues, this paper introduces the Minimum Point Distance Intersection over Union (MPDIoU) [31] as a loss function for bounding box regression. By jointly optimizing the distances of the top-left and bottom-right vertices of the bounding box, it directly constrains the spatial relationship between the predicted box and the ground truth box. The formula is as follows:

(2) $$\mathrm{MPDIoU}=\mathrm{IoU}-\frac{d_{1}+d_{2}}{S}$$

**Figure 6:**
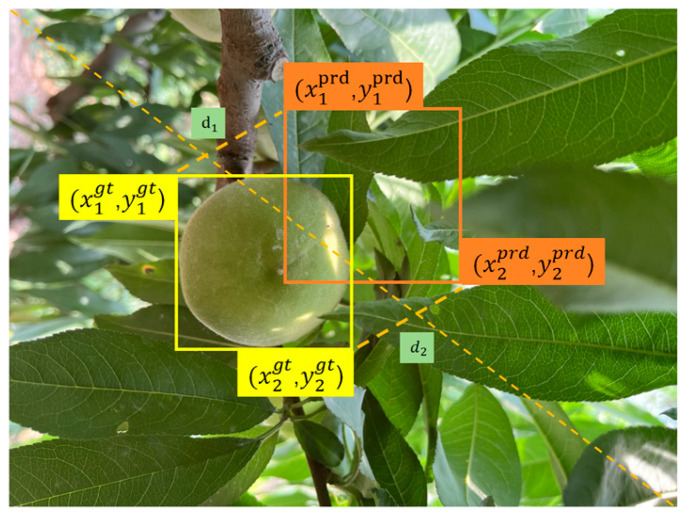
The schematic diagram of the WPDIoU loss function calculation. Yellow: ground truth box. Orange: predicted box.

In the formula, $d_{1}={\left(x_{1}^{prd}-x_{2}^{prd}\right)}^{2}+{\left(y_{1}^{prd}-y_{2}^{prd}\right)}^{2}$ and $d_{2}={\left(x_{1}^{gt}-x_{2}^{gt}\right)}^{2}+{\left(y_{1}^{gt}-y_{2}^{gt}\right)}^{2}$ stand for the squared Euclidean distances of the top-left and bottom-right vertices between the two boxes, respectively. S acts as the normalization factor for image size, which is generally the product of the image’s height and width. This design explicitly incorporates vertex distances into the optimization objective, enabling the predicted box to adjust its center position and aspect ratio simultaneously during the training process. When objects are partially occluded, the joint optimization of vertex distances can more sensitively capture minor deviations of bounding boxes, thus enhancing localization accuracy. The schematic diagram of the WPDIoU loss function calculation is shown in Figure 6.

#### 3.2.5. Enhancing the Neck Network with AMFP

Existing multi-scale fusion methods (e.g., FPN, ASFF, PA-FPN) often employ fixed fusion strategies or coarse-grained spatial weighting, which lack adaptability to dynamic occlusion patterns and illumination-induced scale variations in complex orchard scenes. This rigidity limits their ability to resolve feature ambiguity caused by severe fruit occlusion and background interference. To address this, we propose the AMFP (Adaptive Multi-Scale Fusion Pyramid) module (inspired by spatially-aware feature fusion approaches [32]), which integrates adaptive gating mechanisms with hierarchical feature fusion to a achieve pixel-level, fine-grained control. AMFP dynamically adjusts fusion strategies across high-, medium-, and low-resolution features, enhancing flexibility for complex scenarios, unlike static fusion in standard FPN variants or spatially fixed weighting in ASFF/PA-FPN.

The AMFP first aligns with feature dimensions, as follows: high-level features ($F_{high}$) are downsampled via atrous convolution (kernel = 3 × 3, stride = 2, dilation = 2, padding = 2) to match intermediate-level spatial size. Low-level features ($F_{low}$) are upsampled to intermediate resolution (H × W) via bilinear interpolation. The intermediate feature $F_{mid}$ undergoes channel adjustment using a 1 × 1 convolution with a weight matrix of $W_{m}\in R^{1\times 1\times C_{in}\times C_{out}}$.

Crucially, AMFP introduces channel-wise partitioning and gated fusion to overcome the coarse adaptability of existing methods. Each level’s features are split into 4 sub-blocks (channel count: C/4). The intermediate sub-blocks generate the following gating weights:

(3) $$\alpha^{\left(k\right)}=\sigma (F_{mid}^{\left(k\right)})$$

where σ(·) is the sigmoid activation function, mapping features to the [0, 1] interval as weight values, and $F_{mid}^{\left(k\right)}$ is the k-th (k = 1, 2, 3, 4) sub-block of the intermediate-level feature. Gating weights dynamically adjust the fusion ratio of high- and low-level sub-blocks. The fusion formula is

(4) $$F_{fuse}^{\left(k\right)}=\alpha^{\left(k\right)}⊙F_{high}^{\left(k\right)}+(1-\alpha^{\left(k\right)})⊙F_{low}^{\left(k\right)}$$

This enables pixel-wise adaptive fusion, suppressing occluded regions while enhancing discriminative features, thus addressing the inflexibility of fixed fusion in occlusion-heavy scenarios.

Fused sub-blocks are concatenated channel-wise, processed by a 1 × 1 convolution ($W_{t}\in R^{1\times 1\times C\times C}$), and added to the residual branch of $F_{mid}$ (pre-adjusted by 1 × 1 conv). The final output is derived as follows:

(5) $$F_{out}=BN(Conv2D(F_{fuse};W_{t})+{F^{\prime }}_{mid})$$

(6) $$F_{final}=SiLU(F_{out})$$

**Figure 7:**
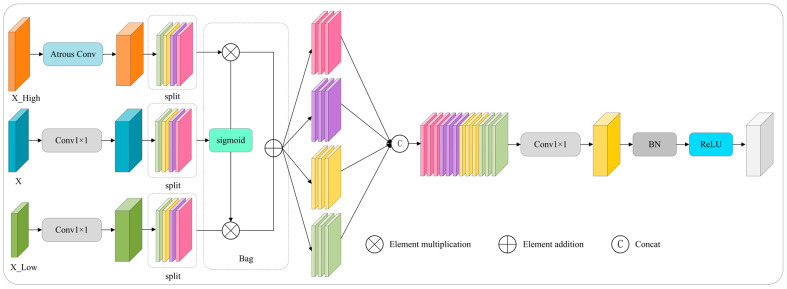
AMFP module structure. ⊗: Element multiplication. ⊕: Element addition. BN: batch normalization operation.

By combining atrous convolution (preserving semantics), pixel-wise gating (resolving occlusion ambiguity), and residual connections (stabilizing gradients), AMFP mitigates the limitations of conventional fusion methods in handling occlusion-driven scale shifts and background noise. The structure is depicted in Figure 7.

## 4. Experimental Platform and Evaluation Matrix

In this study, network training and testing were carried out on a computer equipped with an Intel (R) Xeon (R) Platinum 8474C CPU and an NVIDIA GeForce RTX 4090D GPU, running the Ubuntu operating system. The virtual environment was created via Anaconda 3, utilizing Python 3.10, PyTorch 2.1.0, and CUDA 12.1. During the network training and testing, the computer utilized was equipped with a memory (RAM) capacity of 1.0 TiB (1.0 TB). The training parameter settings were as follows: the input image size was set at 640 × 640, the batch size was set at 64, and the training period was set for 350 epochs, with the Adam optimizer employed for training. The Adam optimizer was configured with an initial learning rate of 0.001 and a weight decay of 0.0005, implementing a cosine annealing scheduler to decay the learning rate to 0.00001 (0.001 × 0.01). During training, model checkpoints were dynamically evaluated after each epoch using mAP@0.5 on the validation set, with the highest-performing checkpoint retained as the final model.

The basic evaluation indicators adopted in this study included precision, recall, and average precision. Precision reflects the reliability of model predictions and is calculated as the ratio of true positives (TP, namely correctly identified peach instances) to the sum of true positives and false positives (FP, namely false alarms). Recall reflects the model’s ability to cover target objects and is calculated as the ratio of true positives to the sum of true positives and false negatives (FN, namely missed instances). Average precision (AP) is obtained by calculating the area under the precision–recall curve, representing a comprehensive measure of detection stability across different recall rate thresholds. In the case of multiple categories, the mean average precision (mAP) is calculated as the average of AP values across all categories. The extended indicators, mAP50 and mAP50-95, employ fixed and dynamic threshold strategies, respectively. The former uses an intersection over union (IoU) of 0.5 as the basic localization accuracy benchmark, while the latter calculates the average value between 0.5 and 0.95 with a step size of 0.05, assessing the model’s robustness to fruit position deviations through multi-threshold integration. The formulas are as follows:

(7) $$Precision=\frac{TP}{TP+FP}$$

(8) $$Recall=\frac{TP}{TP+FN}$$

(9) $$AveragePrecision\left(AP\right)=\int_{0}^{1}P(r)dr$$

(10) $$MeanAveragePrecision\left(mAP\right)=\frac{1}{c}\sum_{i=1}^{c}AP_{I}$$

This evaluation framework integrates indicators of detection accuracy (precision/recall), localization accuracy (mAP-related indicators), and computational efficiency (model parameter scale), forming a quantitative analysis system for agricultural complex scenarios. It provides key criteria for assessing the generalization ability of models in practical applications. Ultimately, this indicator system offers a data-driven decision basis for optimizing fruit detection models through multi-level performance analysis.

## 5. Matrix Experimental Results and Analysis

### 5.1. Ablation Study

To validate the effectiveness of the proposed modules, we conducted ablation experiments on the dataset and evaluated the overall impact of each improved module. The experimental results are shown in Table 2.

This improvement primarily stems from the LSKA module’s ability to provide adaptive large receptive fields. Unlike standard convolutions or fixed-kernel attention mechanisms, LSKA’s decoupled large kernels and dynamic dilation rate effectively capture the multi-scale spatial information of peaches, especially enhancing feature representation for small or partially occluded fruits in dense foliage, which directly contributes to the significant recall (R) and mAP50-95 gains. Further integrating the DMDetect module into the detection head resulted in P, R, mAP50, and mAP50-95 increasing by 0.6%, 2.4%, 1.2%, and 3.7% compared to the baseline, respectively. The DMDetect module addresses a key limitation of the original detection head and standard feature fusion techniques (like basic FPN) in handling severe occlusion. By integrating the DyHead Block, it dynamically combines scale-aware and spatial attention mechanisms within a multi-branch structure. This allows adaptive feature refinement, specifically at the detection stage, significantly improving feature discrimination for occluded peaches and boosting localization accuracy (reflected in mAP50-95) under challenging leaf and branch occlusion scenarios. After introducing the AMFP feature fusion module, P, R, mAP50, and mAP50-95 increased by 3.3%, 0.9%, 1.5%, and 2.6%, respectively. The AMFP module tackles the rigidity inherent in standard FPN, and even adaptive fusion methods like ASFF, when dealing with complex occlusion-induced feature misalignment and scale variance. Its channel-chunking strategy combined with gated attention enables fine-grained, pixel-level adaptive fusion of features from different resolutions. This dynamic weighting mechanism effectively suppresses irrelevant background features and selectively enhances useful information across scales, leading to improved precision (P) and robustness against fruit mutual occlusion and background interference. Finally, when simultaneously applying the SPPF-LSKA, DMDetect, and AMFP modules, P, R, mAP50, and mAP50-95 reached 90.8%, 85.4%, 90%, and 62.7%, respectively, representing improvements of 2.7%, 4.8%, 2.7%, and 7.2% compared to the original YOLOv11n. This synergistic effect demonstrates that the proposed modules collectively address the core limitations of existing building blocks in complex orchard peach detection, as follows: SPPF-LSKA provides robust multi-scale feature extraction with adaptive receptive fields at the backbone level; AMFP offers flexible and fine-grained adaptive fusion in the neck to handle occlusion-induced feature confusion; and DMDetect performs dynamic, occlusion-aware feature refinement at the detection head. Although the model complexity increases (parameters from 2.58 M to 3.56 M, GFLOPs from 6.3 to 8.9, layers from 238 to 310), this increase is justified by the substantial performance gains, particularly the critical 7.2% improvement in mAP50-95, which measures detection quality across IoU thresholds. Crucially, the inference speed still meets the real-time requirements, verifying the effectiveness and practicality of the proposed improvements. This provides robust technical support for intelligent fruit harvesting and yield estimation.

### 5.2. Comparative Experiments on Different Loss Functions

**Figure 8:**
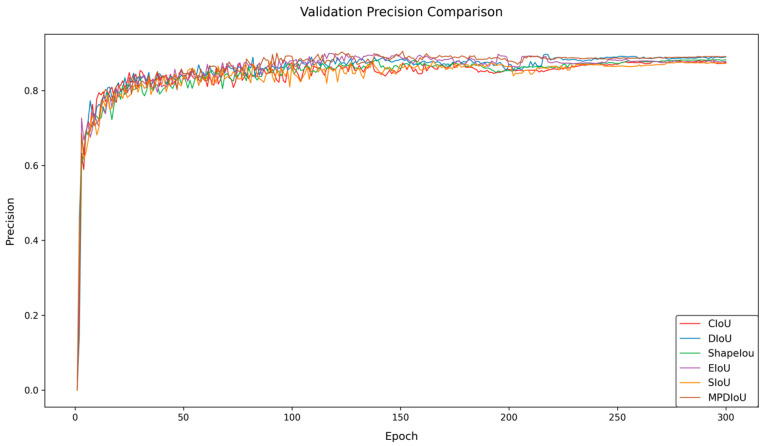
Training accuracy comparison of loss functions.

**Figure 9:**
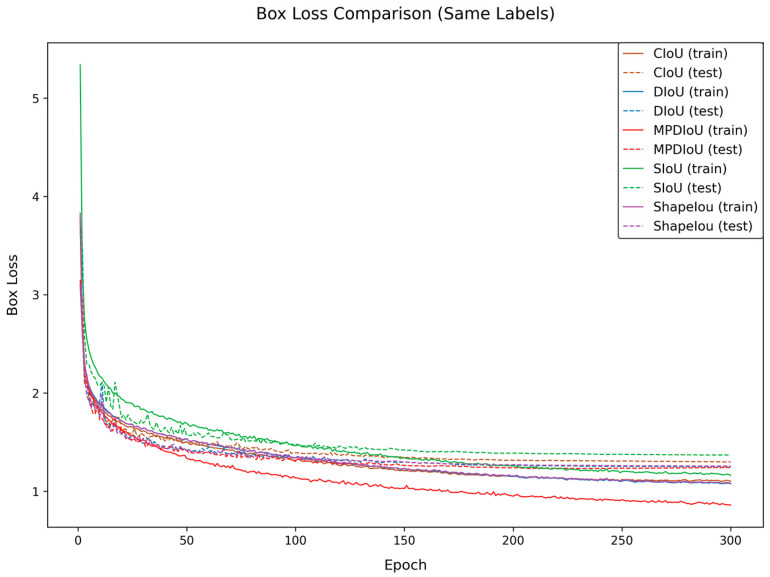
Training loss comparison of loss functions.

To address the challenges of peach detection in complex orchard scenarios, this study conducts comparative experiments by introducing multiple bounding box loss functions to verify their impacts on the model performance. The experiments compare the detection effectiveness of CloU, DloU, EloU, Shapelou, SloU, and MPDIoU. The experimental results are shown in Table 3, and Figure 8 and Figure 9.

The data demonstrate that MPDIoU achieves an optimal performance across all of the following four metrics: precision (P), recall (R), mAP50, and mAP50-95, reaching 88.7%, 81.7%, 88.3%, and 56.2%, respectively. Compared to the baseline model using CloU, MPDIoU improves precision, recall, and mAP50 by 0.6%, 1.1%, and 1.0%, respectively, while mAP50-95 increases by 0.7%. In terms of recall, MPDIoU outperforms DloU and EloU by 0.9% and 0.6%, respectively, and also exhibits superior mAP50-95 compared to other loss functions. Although some loss functions show comparable performance on single metrics (e.g., DloU achieves 88.6% precision), MPDIoU demonstrates significant comprehensive advantages.

Furthermore, Figure 8 and Figure 9 reveal that MPDIoU achieves the highest training accuracy and the lowest loss values during the training process, further validating its comprehensive performance superiority. This study selects MPDIoU as the bounding box regression loss function for the improved model. By jointly optimizing center point distance and aspect ratio, MPDIoU effectively enhances the localization accuracy for overlapping and occluded peaches, laying a solid foundation for subsequent multi-module collaborative optimization.

### 5.3. Comparative Experiments with Other Models

To comprehensively evaluate the performance of the improved model proposed in this study and current state-of-the-art object detection models, SDA-YOLO was compared to various YOLO series models and RT-DETR [33], including YOLOv5n, YOLOv6n, YOLOv8n, YOLOv10n, YOLOv11n, YOLOv12n, RT-DETR-l, and RT-DETR-resnet50.

The YOLO series represents the most popular single-stage object detection models in recent years, while RT-DETR is a novel transformer-based object detection model renowned for its outstanding performance and flexibility, particularly excelling in complex scenarios. Through comparisons to these diverse models, this paper conducts a more comprehensive assessment of the performance advantages and limitations of the SDA-YOLO model. The comparative experimental results are shown in Table 4.

The experimental results demonstrate that although SDA-YOLO does not possess the smallest number of parameters or model size, it exhibits a superior detection performance compared to other models. Its precision, recall, mAP50, and mAP50-95 reach 90.8%, 85.4%, 90%, and 62.7%, respectively, representing the highest values among all evaluated models. Notably, while maintaining significantly fewer parameters and lower computational complexity than the RT-DETR series models, SDA-YOLO surpasses RT-DETR in detection performance. Furthermore, it achieves an end-to-end FPS of 75.8, meeting the demands for real-time agricultural applications, which demonstrates its excellent balance between performance and efficiency.

**Figure 10:**
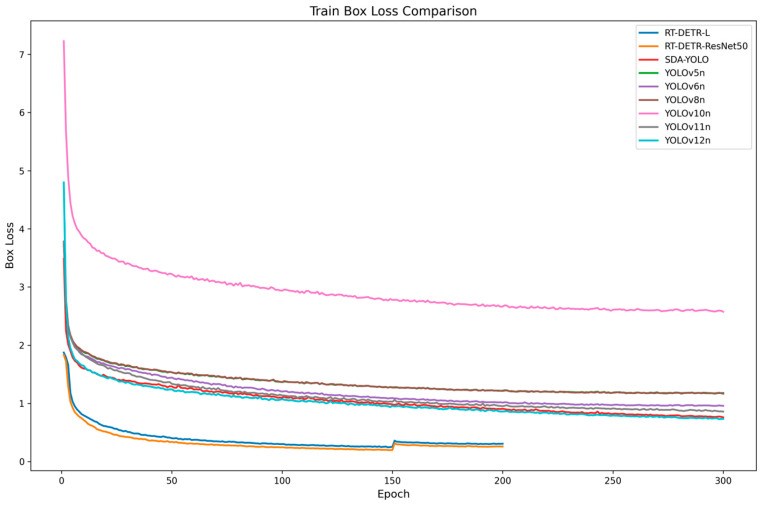
Comparative Training Box Loss Curves of Peach Detection Models.

To further analyze the training dynamics that are critical for field deployment, Figure 10 presents comparative training loss curves. The RT-DETR models (both L and ResNet50 variants) achieved the fastest convergence, stabilizing at approximately 200 epochs due to early stopping, with final loss values of 0.5–0.6. Among YOLO series models, the convergence speed varied substantially, as follows: YOLOv10n exhibited the slowest convergence and highest final loss (~3.0), suggesting training instability. Other models (YOLOv5n, YOLOv6n, YOLOv8n, YOLOv11n, YOLOv12n) demonstrated intermediate convergence speeds, with final losses of 1.2–1.8. SDA-YOLO converged at a rate comparable to YOLOv12n while maintaining stable loss values within this optimal range. This convergence efficiency, combined with its architectural innovations, enhances operational robustness in complex orchard environments.

### 5.4. Comparative Experiments with Different Attention Mechanisms Integrated into the SPPF Module

To investigate the impact of different attention mechanisms on detection model performance, this study conducts comparative experiments by embedding various attention mechanisms into the SPPF module. Five attention mechanisms—BAM [34], EMA [35], SE [36], AIFI [33], and LSKA—are selected to construct improved models for performance comparison. Detailed results are shown in Table 5.

The data in Table 5 demonstrate that among the five introduced attention mechanisms, the model with LSKA achieves the optimal performance in accuracy and mAP50-95, reaching 89.0% and 58.8%, respectively. Compared to the models using the AIFI, BAM, EMA, and SE mechanisms, the LSKA mechanism improves accuracy by 1.1%, 0.02%, 0.49%, and 0.70%, while enhancing mAP50-95 values by 0.6%, 1.7%, 1.0%, and 1.7%, respectively. Regarding mAP50, although LSKA shows only a 0.1% gap compared to the best-performing SE mechanism, it surpasses SE by 0.1% in accuracy and 1.7% in mAP50-95, fully demonstrating the superior comprehensive detection performance of the LSKA mechanism. In recall rate, LSKA lags behind the top-performing EMA mechanism by 1.2%, but exceeds EMA by 2.8% in accuracy, 0.2% in mAP50, and 1.0% in mAP50-95, indicating that LSKA maintains high localization accuracy while achieving a more balanced detection capability for complex targets.

**Figure 11:**
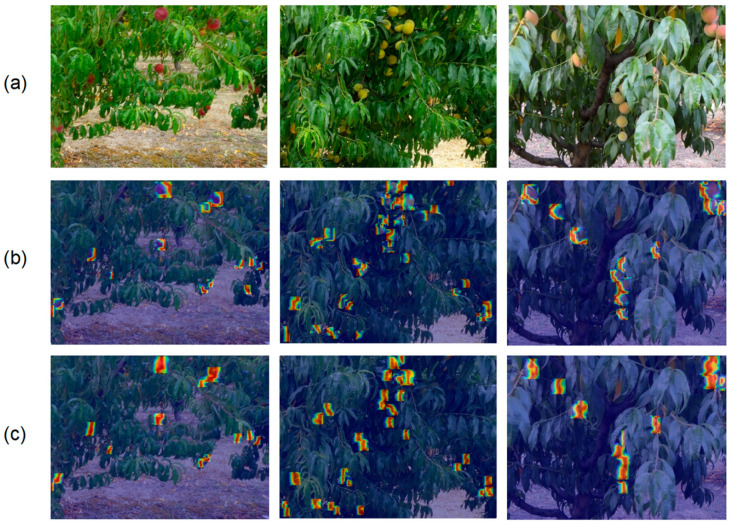
SPPF-LSKA heatmap comparison. (**a**) Original image. (**b**) SPPF heatmap. (**c**) SPPF-LSKA heatmap. Warmer colors denote regions with higher attention weights assigned by the model, indicating stronger focus.

To further verify the superiority of LSKA, a heatmap visualization analysis of the model’s attention focus is conducted, with the results shown in Figure 11. The heatmaps clearly reveal that the SPPF module with LSKA can more precisely capture critical features in target regions while effectively filtering out interference from complex backgrounds in orchard images. In conclusion, the introduction of the LSKA attention mechanism into the SPPF module significantly enhances the model’s selective focusing capability on target features, thereby improving the overall detection performance.

### 5.5. Robustness Testing

To verify the model’s generalization capability in complex natural environments, this study conducted systematic robustness tests targeting common meteorological conditions in orchard scenarios. The test data were generated by augmenting the original set of 97 test images. For each meteorological condition, a corresponding test subset was created by doubling the size through data augmentation.

**Figure 12:**
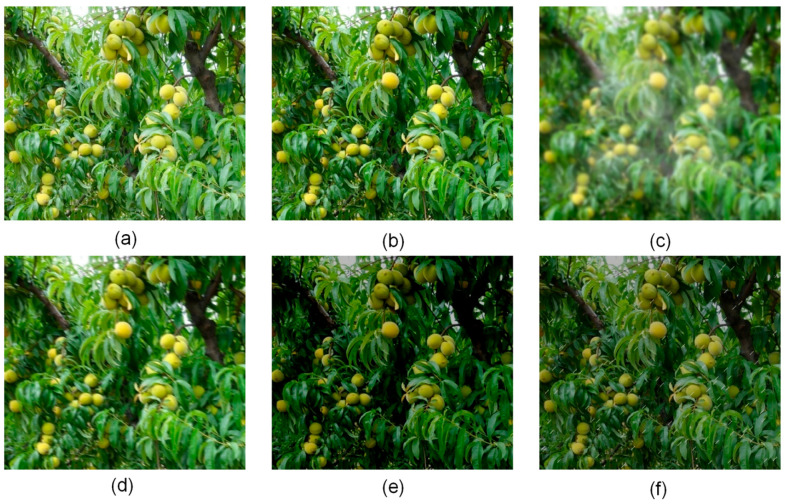
Visualization of Weather Condition Augmentation Effects. (**a**) Base. (**b**) Low_light. (**c**) Fog. (**d**) Motion_blur. (**e**) Overcast. (**f**) Rain.

For the foggy scenario enhancement, a light fog effect was applied (fog coefficient: 0.03–0.10, transparency coefficient: 0.05) to avoid completely occluding fruit features. This parameter configuration aligns with the visibility characteristics of actual orchard morning fog scenarios, effectively reducing visibility while preserving fruit identifiability. It also accounts for the practical constraint that heavy fog environments are unsuitable for mechanical operations. The cloudy day enhancement simulated illumination attenuation by reducing the brightness (−0.20 to −0.10) and contrast (−0.15 to −0.05), combined with CLAHE enhancement (grid size: 8×8, contrast threshold: 2.0) to maintain detail visibility and prevent feature loss due to insufficient light. The rainy day enhancement simulated drizzle effects using short rain streaks (length: 8 pixels) and thin rain streaks (width: 1 pixel), with random inclination angles within 15 degrees and with slight blurring (blur value: 1). This parameter design, distinct from settings for heavy rain scenarios, corresponds to the practical condition that light rain does not impede picking operations. The motion blur enhancement employed a limited blur range (5–10 pixels) to simulate equipment vibration, ensuring the fruit contours remained identifiable and avoiding target non-recognition caused by severe shaking. The low-light environment enhancement simulated dusk light attenuation via gamma value adjustment (70–90), supplemented by a slight Gaussian blur (1–3 pixels) to simulate low-light imaging noise. All augmentation parameters were controlled within mild to moderate ranges, reflecting the feasibility boundaries of actual orchard operations; furthermore, excessively adverse conditions (such as heavy rain, thick fog, or severe vibration) are inherently unsuitable for mechanical operation, and they fall outside of the model’s reasonable application scenarios. Figure 12 displays sample images after the aforementioned augmentations.

Using the augmented datasets, tests were conducted with YOLOv11n and SDA-YOLO. The experimental results are shown in Table 6 and Table 7. In low-light environments, SDA-YOLO’s precision (P) increased from 90.8% in the baseline scenario to 94.1%, and mAP50 increased from 90.0% to 91.9%. This indicates that its feature discrimination capability, enhanced through gamma correction and noise simulation, effectively overcomes illumination attenuation. In contrast, YOLOv11n’s P value decreased by 2.4% (from 88.1% to 85.7%), validating the optimization effect of the AMFP module for low-light feature fusion. For foggy scenarios, SDA-YOLO’s mAP50 decreased to 75.1% (baseline: 90.0%). Although this outperformed YOLOv11n’s 71.3%, its P value decreased by 2.0% (from 90.8% to 88.8%). This reveals the model’s continued sensitivity to feature occlusion caused by fog, consistent with the physical limitations morning fog imposes on vision systems in actual operations, necessitating further optimization through subsequent feature enhancement. Under the dynamic blur simulating equipment vibration, SDA-YOLO demonstrated a robust performance, achieving a P value of 91.3% (higher than the baseline 90.8%), with mAP50 decreasing by only 2.3% (from 90.0% to 87.7%). Leveraging the DyHead module’s motion feature extraction capability, the model maintained good target identifiability within the limited blur range. In cloudy environments, SDA-YOLO’s P value and mAP50 decreased to 85.1% and 86.0%, respectively. However, it still outperformed YOLOv11n (P: 79.2%, mAP50: 83.3%). The synergistic enhancement strategy combining CLAHE and the brightness adjustment effectively mitigated feature degradation due to insufficient light, although cloud cover still exerted a slight negative impact on detection accuracy. For rainy scenarios, SDA-YOLO exhibited the best adaptability, maintaining a high P value of 91.1% and increasing mAP50 to 91.2% (baseline: 90.0%). The drizzle simulation validated the MPDIoU loss function’s effectiveness in optimizing the localization of occluded targets under rain streak interference, meeting the technical requirements for light rain operation scenarios.

SDA-YOLO maintained efficient inference capabilities under most meteorological conditions. Notably, its FPS in rainy (105.3 FPS) and low-light (92.6 FPS) scenarios was significantly higher than in the baseline scenario (75.8 FPS). This indicates that the Adaptive Multi-Level Feature Fusion mechanism (AMFP) can optimize the computational load under specific environmental conditions.

The experiments demonstrate that SDA-YOLO, by integrating the SPPF-LSKA multi-scale feature enhancement, MPDIoU localization optimization, and the DyHead occlusion discrimination module, achieved improvements in both P value and mAP50 under low-light, rainy, and dynamic blur scenarios. This significantly strengthens its adaptability to complex orchard meteorology. Although there is room for optimization in foggy scenarios, its overall performance validates the effectiveness of the improved architecture in real-world operation scenarios, providing a more reliable visual perception solution for intelligent orchard equipment.

### 5.6. Visualization of Detection Results

**Figure 13:**
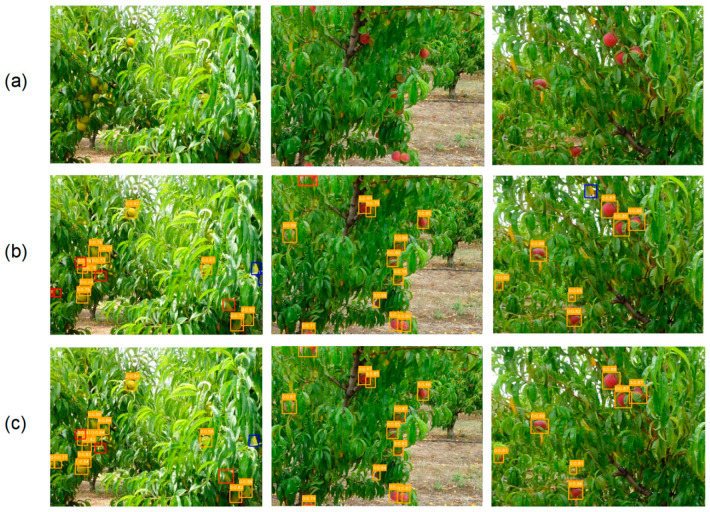
Visualization of detection results comparison. (**a**) Original images. (**b**) YOLOv11n model. (**c**) SDA-YOLO model.

To visually demonstrate the SDA-YOLO model’s performance in peach fruit detection under orchard conditions, three representative images of different peach varieties were selected from the dataset, showing part of the detection results, as shown in Figure 13. Row (a) shows the original images of the three peach varieties. Row (b) presents the YOLOv11n model’s detection results. Row (c) presents the SDA-YOLO model’s detection results. In rows (b) and (c), missed detections are marked with red boxes, and false detections are marked with blue boxes.

Compared to column 1, it can be found that SDA-YOLO can detect smaller and more occluded peach fruits. YOLOv11n is relatively weak in detecting these small and occluded fruits, which shows that SDA-YOLO has a stronger feature extraction and expression ability in dealing with occluded and small-scale targets. Compared to column 2, it can be obviously found that SDA-YOLO still has advantages in detecting peach fruits with large occlusion areas. It can more accurately locate the fruits occluded by leaves and branches, with a higher detection accuracy and fewer missed and false detections. This is attributed to the DyHead Block-based DMDetect module and AMFP feature fusion module of SDA-YOLO. The combination of the multi-branch dynamic detection structure and adaptive mechanism enhances the feature discrimination of occluded targets and multi-scale feature fusion in complex backgrounds. Compared to column 3, it can be found that SDA-YOLO not only focuses on the color of peaches, but also emphasizes their texture features. In complex backgrounds with similar fruit colors, it reduces false detection rates by accurately capturing texture features. This is mainly due to the multi-scale feature expression advantages brought by the SPPF-LSKA fusion module in the backbone network and the overall network’s comprehensive feature analysis ability.

SDA-YOLO achieves strong peach detection in complex orchards, but Figure 12c reveals minor false negatives (FNs) and false positives (FPs). For example, a heavily occluded fruit with minimal visibility (lower-left, second column) was missed (FN). Conversely, a background patch resembling a peach in texture/color was falsely detected (FP, blue box, third column). This highlights challenges with extreme occlusion and high-similarity background interference.

False negatives (FNs) likely result from insufficient visible information under severe occlusion, which hinders the capture of discriminative features. Conversely, false positives (FPs) may stem from local background regions (e.g., leaves or soil under dappled light) sharing texture or color characteristics with peaches at specific scales, particularly when the model relies heavily on such texture cues. Future efforts will therefore focus on reducing both types of errors through several key strategies, as follows: enhancing attention mechanisms to better leverage residual visible parts of occluded objects; developing approaches like adversarial training or feature decoupling to mitigate confusion from high-similarity backgrounds; optimizing bounding box loss functions (e.g., MPDIoU) specifically for occluded targets; and exploring the integration of additional cues, such as temporal information from video sequences or complementary data from modalities like near-infrared imaging.

## 6. Discussion

### 6.1. Model Performance and Limitations

The SDA-YOLO model proposed in this study demonstrates significant efficacy in the task of peach fruit detection within complex orchard environments. While maintaining a lightweight design, the model achieves high detection accuracy and real-time inference speed. The experimental results validate that the synergistic integration of the SPPF-LSKA module, DMDetect detection head, AMFP feature pyramid, and MPDIoU loss function effectively addresses core challenges present in complex orchard scenes, including multi-scale variations, severe occlusion, and background interference. Crucially, SDA-YOLO retains real-time inference capability despite the introduction of adaptive mechanisms and the maintenance of high accuracy, reflecting a good balance between efficiency and precision in the model.

However, this study has several limitations that constrain the model’s generalization capability. Specifically, although the public dataset employed encompasses three peach varieties, it is insufficient to represent the diversity of fruit species, the complexity of orchard environments, and the wide range of environmental variations (e.g., differing lighting conditions, occlusion levels, shooting distances, angles, and weather conditions) encountered in real-world scenarios. These limitations stem from practical constraints in data collection, such as seasonal restrictions, labor-intensive annotation requirements, and the complexity of capturing all possible orchard variations. Consequently, the model’s robustness and generalization ability when encountering unseen scenarios may be compromised. To address this, future work necessitates validation on additional or independently collected datasets to rigorously assess model performance.

Regarding specific constraints, the data acquisition conditions failed to adequately encompass/complex orchard lighting conditions (e.g., front lighting, backlighting, strong light, weak light), diverse occlusion scenarios (e.g., large-scale dense fruit occlusion, partial occlusion by leaves), varying shooting distances (close- and long-range), multiple shooting angles, diverse weather conditions (sunny, cloudy, overcast), and background interference. These data limitations partially constrain the model’s robustness and generalization ability. Additionally, constrained by computational resources and time constraints, this study did not conduct multiple repeated experiments to provide statistical significance tests. Future works will validate the statistical robustness of the results on a larger scale of experiments and explore methods for calculating confidence intervals. Regarding specific performance limitations, the current detection method exhibits limitations in identifying fruit under high occlusion rates, leading to missed detections in cases of severe occlusion. Additionally, the model’s recognition performance fluctuates across different peach varieties, indicating that its generalization stability requires further enhancement.

Future works will focus on the following directions to mitigate these limitations and strengthen validation. First, we will collect peach fruit data covering a broader range of varieties, maturity stages, and diverse environmental conditions (particularly the following aforementioned complex scenarios: lighting, occlusion, angle, weather) to construct a more representative dataset, thereby improving model robustness and generalization. This will explicitly include plans to evaluate the model on datasets from independent sources to validate its applicability across different scenarios. Second, we will conduct in-depth algorithmic optimization targeting the challenge of highly occluded fruit detection, exploring more effective feature extraction and fusion strategies. Finally, building upon this study’s detection framework, we will extend the research to conduct a precise maturity classification of peach fruit in complex orchard environments, further exploring the method’s application potential in intelligent orchard management. The demonstrated high accuracy and real-time performance of SDA-YOLO, coupled with its lightweight design (3.55 M parameters, 8.9 GFLOPs), suggests its potential for integration into cost-effective edge computing systems. This lays a foundation for practical applications such as automated yield estimation and robotic harvesting, which could significantly reduce labor costs associated with manual counting and picking. We emphasize that such future validation is not merely supplementary, but fundamental for translating the research findings into practical applications.

### 6.2. Deployment Requirements, Challenges, and Broader Implications

The high accuracy, real-time performance, and lightweight design of SDA-YOLO (3.55 M parameters, 8.9 GFLOPs) demonstrate its potential for integration into cost-effective edge computing systems. This establishes a foundation for practical applications like automated yield estimation and robotic harvesting, promising significant reductions in labor costs associated with manual counting and picking. However, deploying such models in real-world orchard environments introduces specific requirements and challenges. Computational constraints on embedded platforms (e.g., harvesting robots, drones, mobile yield estimation devices) necessitate further optimization. While currently lightweight, techniques like quantization (reducing numerical precision of weights/activations), pruning (removing redundant connections), and hardware-aware neural architecture search could further reduce model size and inference latency, enhancing suitability for resource-limited edge devices. Environmental factors like variable lighting, dust, vibration, and temperature fluctuations demand robust hardware enclosures and potentially adaptive software strategies beyond the model’s inherent robustness. Power consumption remains a critical factor for battery-operated field devices, requiring careful trade-offs between detection frequency, model complexity, and energy efficiency.

Quantifying its potential agricultural impact, SDA-YOLO’s achieved precision (90.8%) and recall (85.4%) on the test set represent a significant step towards reliable automated fruit counting and localization. Applied to yield estimation, this accuracy level could substantially improve harvest planning and resource allocation. For robotic harvesting, while the model provides essential detection capability, effective integration with robotic arms for grasping and detaching fruits under occlusion remains a complex challenge, requiring further mechatronic and control system development. The core innovations—the AMFP module for adaptive multi-scale fusion, DMDetect for enhanced occlusion handling, and MPDIoU for improved localization—are not inherently peach-specific. The architectural principles could be adapted to detect other fruits (e.g., apples, citrus, mangoes) in similar complex agricultural settings. Replicating the approach primarily requires retraining the model on annotated datasets of the target fruit species and potentially fine-tuning the hyperparameters. The modular design facilitates this transfer, as the backbone, neck (AMFP), and detection head (DMDetect) components could be reused or minimally modified. Future validation on diverse datasets and deployment scenarios is fundamental for translating the research findings into practical applications and realizing the full economic potential of automated peach detection and management systems in agriculture.

## 7. Conclusions and Future Work

In complex orchard scenarios, peach fruit detection faces multiple challenges. This study proposes SDA-YOLO, an improved YOLOv11n-based peach detection method. On one hand, in the backbone network, we construct an SPPF-LSKA fusion module by embedding LSKA into the SPPF module, leveraging adaptive receptive field adjustment and the spatial information capture capability of large kernel convolution to enhance the multi-scale feature representation for peach targets. On the other hand, we introduce an MPDIoU-based bounding box regression loss function to replace CIoU, optimizing the center point distance and aspect ratio to improve the localization accuracy for overlapping and occluded peaches. Meanwhile, we integrate DyHead Block into the detection head to construct a DMDetect module, forming a multi-branch dynamic detection structure that enhances feature discrimination for small targets and occluded objects in complex backgrounds through scale-aware and spatial attention mechanisms. Additionally, to address feature fusion issues in multi-scale target detection, we propose an AMFP module that combines adaptive mechanisms, with multi-scale feature fusion and pyramid structures, dynamically adjusting fusion strategies across different hierarchical features to enhance model flexibility in processing complex features. Experiments demonstrate that SDA-YOLO outperforms YOLOv11n in accuracy, recall, mAP@0.5, and mAP@0.5:0.95 metrics, validating its robustness and providing technical support for intelligent fruit picking and yield estimation. The maintained real-time inference speed (75.8 FPS) and lightweight architecture indicate the feasibility of deploying SDA-YOLO on affordable edge devices, potentially lowering the barrier to adoption for technologies aimed at automating orchard tasks and reducing operational costs. In future research, we will collect more samples from different peach varieties, retrain the model to learn broader peach fruit characteristics, and focus on maturity classification research in complex orchard environments to further expand the method’s application scope and value. These efforts are crucial steps towards realizing the full economic potential of automated peach detection and management systems in agriculture.

## Figures and Tables

**Table 1:** The number of peach fruits.

Dataset	Number of Images	Number of Peach
Training set	4032	24,320
Validation set	192	1146
Test set	97	623

**Table 2:** Ablation study results.

SPPF-LSKA	DMDetect	AMFP	Parameters	GFLOPs	P	R	mAP50	mAP50-95	Layers
x	x	x	2,582,347	6.3	0.881	0.806	0.873	0.555	238
√	x	x	2,855,243	6.5	0.890	0.826	0.888	0.588	244
x	√	x	3,099,207	7.4	0.887	0.830	0.885	0.592	273
x	x	√	2,679,627	7.4	0.914	0.815	0.888	0.581	269
√	√	x	3,372,103	7.7	0.884	0.840	0.891	0.592	279
√	x	√	2,952,523	7.6	0.882	0.817	0.881	0.581	275
x	√	√	3,283,079	8.7	0.907	0.804	0.885	0.584	304
√	√	√	3,555,975	8.9	0.908	0.854	0.900	0.627	310

**Table 3:** Comparative experimental results of different loss functions.

Bbox_Loss	P	R	mAP50	mAP50-95
CIoU	0.881	0.806	0.873	0.555
DIoU	0.886	0.808	0.883	0.557
EIoU	0.881	0.811	0.881	0.551
ShapeIoU	0.886	0.804	0.878	0.560
SIoU	0.870	0.811	0.881	0.553
MPDIoU	0.887	0.817	0.883	0.562

**Table 4:** Comparison experiments with other models.

Model	Parameters	GFLOPs	P	R	mAP50	mAP50-95	Layers	FPS
Yolov5n	2,181,859	5.8	0.876	0.809	0.874	0.560	211	100.0
Yolov6n	4,155,123	11.5	0.899	0.811	0.871	0.584	160	84.7
Yolov8n	2,684,563	6.8	0.864	0.805	0.879	0.563	186	78.7
Yolov10n	2,265,363	6.5	0.820	0.790	0.858	0.548	229	111.1
Yolov11n	2,582,347	6.3	0.881	0.806	0.873	0.555	238	89.3
Yolov12n	2,508,539	5.8	0.856	0.832	0.877	0.580	376	73.0
RTDETR-1	31,985,795	103.4	0.900	0.814	0.884	0.594	502	50.0
RTDETR-resnet50	41,936,739	125.6	0.897	0.839	0.886	0.603	440	42.6
SDA-YOLO	3,555,975	8.9	0.908	0.854	0.900	0.627	310	75.8

**Table 5:** Comparative experiments with different attention mechanisms integrated into the SPPF module.

Model	Parameters	GFLOPs	P	R	mAP50	mAP50-95	Layers
SPPF-AIFI	3,207,883	6.6	0.879	0.822	0.884	0.582	243
SPPF-BAM	2,662,412	6.3	0.888	0.809	0.883	0.571	269
SPPF-EMA	2,623,563	6.6	0.862	0.834	0.886	0.578	246
SPPF-SE	2,615,115	6.3	0.889	0.811	0.889	0.571	245
SPPF-LSKA	2,855,243	6.5	0.890	0.826	0.888	0.588	244

**Table 6:** Performance Comparison of YOLOv11n under Baseline and Augmented Weather Conditions.

Yolov11n	P	R	mAP50	mAP50-95	FPS
Base	0.881	0.806	0.873	0.555	89.3
Low_light	0.857	0.839	0.889	0.616	71.9
Fog	0.874	0.522	0.713	0.359	66.2
Motion_blur	0.883	0.774	0.860	0.513	68.0
Overcast	0.792	0.777	0.833	0.492	67.1
Rain	0.861	0.825	0.886	0.599	79.4

**Table 7:** Performance Comparison of SDA-YOLO under Baseline and Augmented Weather Condition.

SDA-YOLO	P	R	mAP50	mAP50-95	FPS
Base	0.908	0.854	0.900	0.627	75.8
Low_light	0.941	0.839	0.919	0.689	92.6
Fog	0.888	0.583	0.751	0.419	76.3
Motion_blur	0.913	0.788	0.877	0.562	86.2
Overcast	0.851	0.774	0.860	0.566	77.5
Rain	0.911	0.839	0.912	0.677	105.3

## Data Availability

The peach fruit dataset utilized in this study can be found at the following link: https://github.com/PeachDataset/Dataset (accessed on 1 April 2025). The source code of SDA-YOLO, the training and evaluation scripts, as well as detailed instructions for reproducing experiments, can be obtained from the corresponding author upon request.
